# Synthesis and crystal structures of two 1,3-di(alk­yloxy)-2-(methyl­sulfan­yl)imidazolium tetra­fluorido­borates

**DOI:** 10.1107/S2056989020003643

**Published:** 2020-03-17

**Authors:** Thomas Gelbrich, Martin Lampl, Gerhard Laus, Volker Kahlenberg, Hubert Huppertz, Herwig Schottenberger

**Affiliations:** a University of Innsbruck, Institute of Pharmacy, Innrain 52, 6020 Innsbruck, Austria; b University of Innsbruck, Faculty of Chemistry and Pharmacy, Innrain 80-82, 6020 Innsbruck, Austria; c University of Innsbruck, Institute of Mineralogy and Petrography, Innrain 52, 6020 Innsbruck, Austria

**Keywords:** conformation, imidazole, methyl­sulfanyl heterocycle, tetra­fluorido­borate, crystal structure

## Abstract

Two salts were prepared by methyl­ation of the respective imidazoline-2-thione at the sulfur atom, using Meerwein’s salt (tri­methyl­oxonium tetra­fluorido­borate) in CH_2_Cl_2_. 1,3-Dimeth­oxy-2-(methyl­sulfan­yl)imidazolium tetra­fluorido­borate (**1**), C_6_H_11_N_2_O_2_S^+^·BF_4_^−^, displays a *syn* conformation of its two meth­oxy groups relative to each other whereas the two benz­yloxy groups present in 1,3-dibenz­yloxy-2-(methyl­sulfan­yl)imidazolium tetra­fluorido­borate (**2**), C_18_H_19_N_2_O_2_S^+^·BF_4_^−^, adopt an *anti* conformation. In the mol­ecules of **1** and **2**, the methyl­sulfanyl group is rotated out of the plane of the respective heterocyclic ring. In both crystal structures, inter­molecular inter­actions are dominated by C—H⋯F—B contacts, leading to three-dimensional networks. The tetra­fluorido­borate counter-ion of **2** is disordered over three orientations (occupancy ratio 0.42:0.34:0.24), which are related by rotation about one of the B—F bonds.

## Abstract 2

In both structures, the alk­yloxy and methyl­sulfanyl groups are rotated out of the plane of the respective heterocyclic ring.

## Chemical context   

2-(Methyl­thio)­imidazolium salts have attracted great inter­est because of their reactive properties. Compounds belonging to this class can be converted into important derivatives with useful biological activity, *i.e.* as anti-filarial agents (Link *et al.*, 1990 ▸). Furthermore, they have been used as precursors for the synthesis of remote *N*-heterocyclic carbene complexes (*r*NHC) (Patel *et al.*, 2018 ▸), as tunable alkyl­ating reagents (Guterman *et al.*, 2018 ▸) or as coupling reagents for the formation of bis­(2-imidazol­yl)methyl­ium salts (Kuhn *et al.*, 1993 ▸; Fürstner *et al.*, 2008 ▸).

The S-methyl­ation of thio­nes, typically with methyl iodide or Meerwein’s salt (tri­methyl­oxonium tetra­fluorido­borate), is straightforward. The title compounds **1** and **2** were prepared by methyl­ation of 1,3-di­meth­oxy­imidazoline-2-thione (Laus *et al.*, 2013 ▸) and 1,3-di(benz­yloxy)imidazoline-2-thione (Laus *et al.*, 2016 ▸), respectively, using Meerwein’s salt in CH_2_Cl_2_. An analogous procedure was applied by Williams *et al.* (1994 ▸) for the synthesis of the classic 1,3-dimethyl-2-(methyl­sulfan­yl)imidazolium iodide.

## Structural commentary   

**Figure 1:**
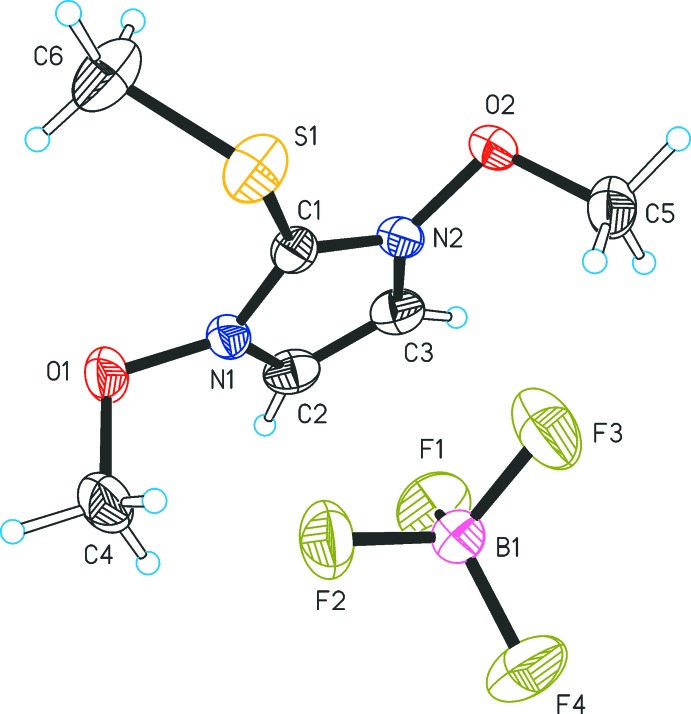
The ion pair structure of methyl­sulfanyl salt **1**, showing displacement ellipsoids drawn at the 50% probability level and hydrogen atoms drawn as spheres of arbitrary size.

In the organic cation of **1**, the two meth­oxy groups adopt a *syn* conformation relative to each other, and the methyl­sulfanyl group is *anti* to each of the meth­oxy groups (Fig. 1 ▸). In contrast, the structurally related mol­ecule of 1,3-di­meth­oxy­imidazoline-2-thione displays an *anti* conformation of its meth­oxy groups (Laus *et al.*, 2013 ▸). The two N—OMe fragments of **1** form dihedral angles with the mean plane of the imidazole ring of 82.3 (2)° (for the ring involving O1 and C4) and of 76.8 (1)° (for the ring involving O2 and C5). The methyl­sulfanyl group (S1–C6) is rotated out of the heterocyclic plane and forms a dihedral angle of 62.5 (1)° with the mean plane of the heterocycle defined by atoms N1, C1, N2, C2, and C3.
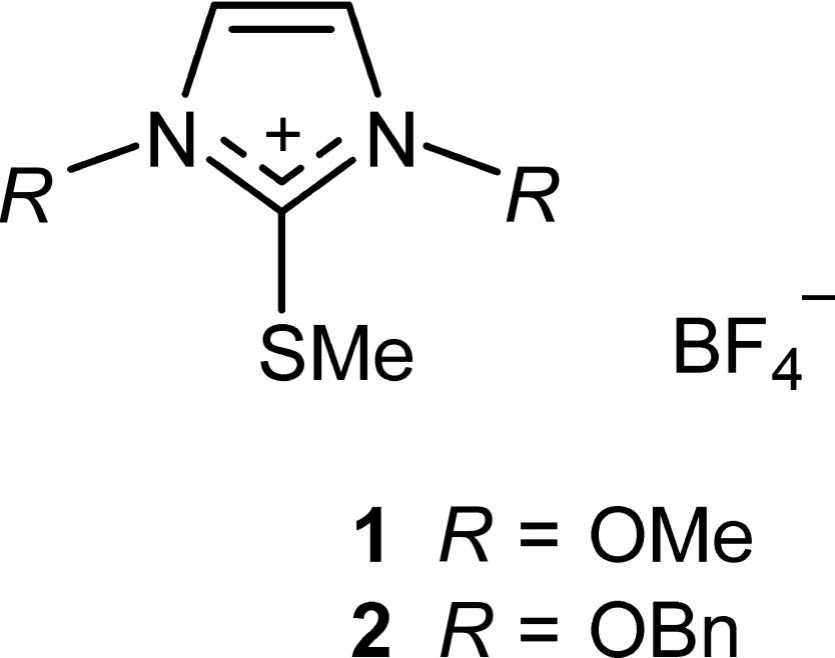


**Figure 2:**
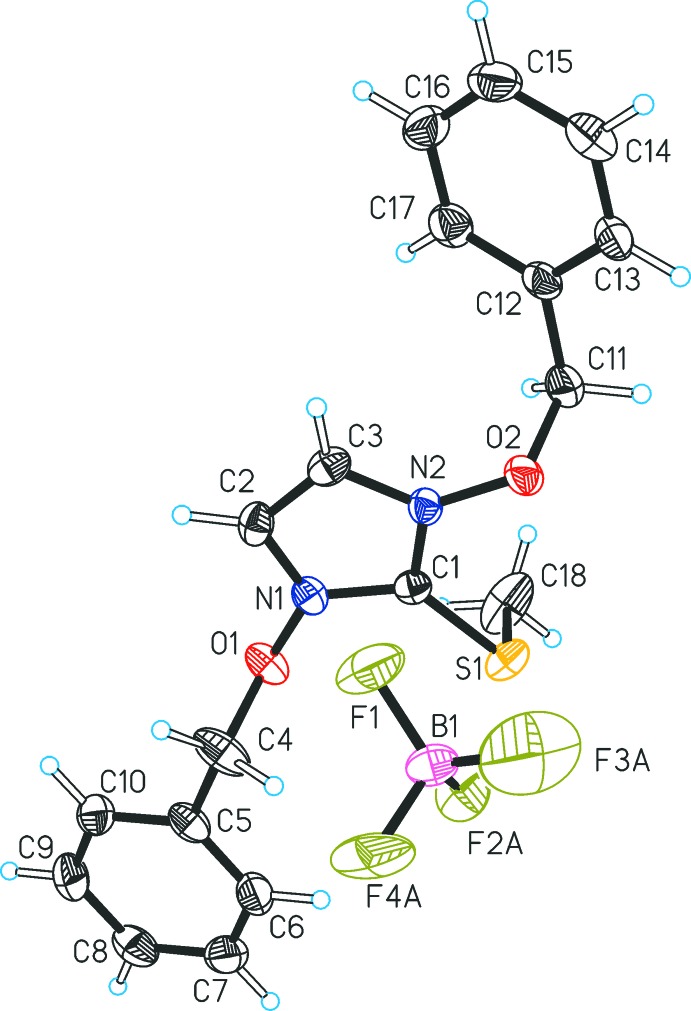
The ion pair structure of methyl­sulfanyl salt **2**, showing displacement ellipsoids drawn at the 50% probability level and hydrogen atoms drawn as spheres of arbitrary size. Only one of the three different orientations of the disordered BF_4_^−^ anion is shown.

**Figure 3:**
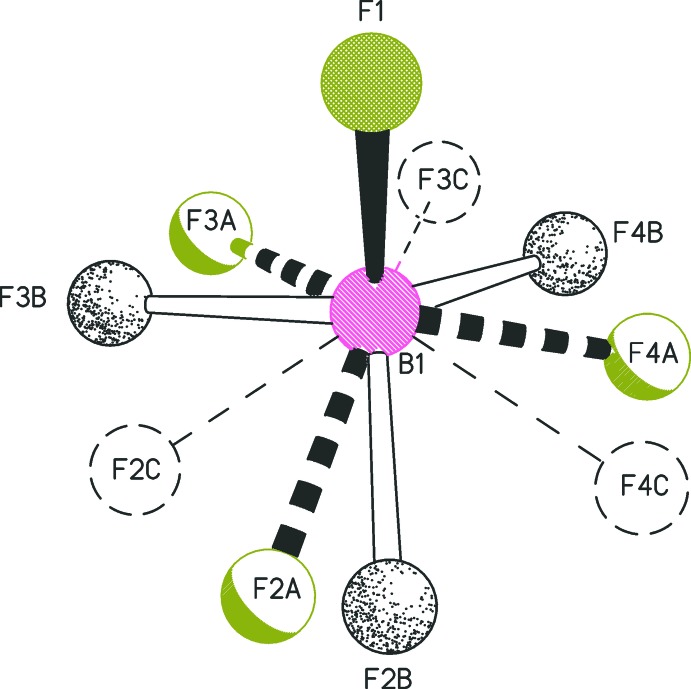
Disorder of the tetra­fluorido­borate anion in the structure of **2**. The disorder components *A* (F2*A*, F3*A*, F4*A*), *B* (F2*B*, F3*B*, F4*B*) and *C* (F2*C*, F3*C*, F4*C*) are related by a rotation about the B1—F1 bond.

Similar to **1**, the methyl­sulfanyl group (S1–C18) of **2** is rotated out of the plane of the heterocycle and forms a dihedral angle of 78.6 (1)° with the mean plane defined by the imidazole ring atoms (N1, C1, N2, C2, C3). The arrangement of the two benz­yloxy moieties in the cation of **2** relative to each other is *anti* (Fig. 2 ▸). They adopt distinct conformations, which is illustrated by the different values of the torsion angles N1—O1—C4—C5 = −174.2 (2)° and N2—O2—C11—C12 = 95.5 (2)°. The two benzene ring planes are inclined by 17.34 (9)° for C5–C10 and by 30.6 (1)° for C12–C17 relative to the plane of the central heterocycle. The tetra­fluorido­borate counter-ion of **2** is disordered over three orientations (occupancy ratio 0.42:0.34:0.24), which are related by a rotation about the B1—F1 bond (Fig. 3 ▸).

The _heterocycle_C—S bond lengths [1.722 (2) and 1.721 (3) Å for **1** and **2**, respectively] determined in this study are in good agreement with the mean value (1.735 Å) calculated from 82 pertinent C—S distances compiled in the Cambridge Structural Database (selection criterion *R*_1_ < 0.10; Groom *et al.*, 2016 ▸).

## Supra­molecular features   

**Figure 4:**
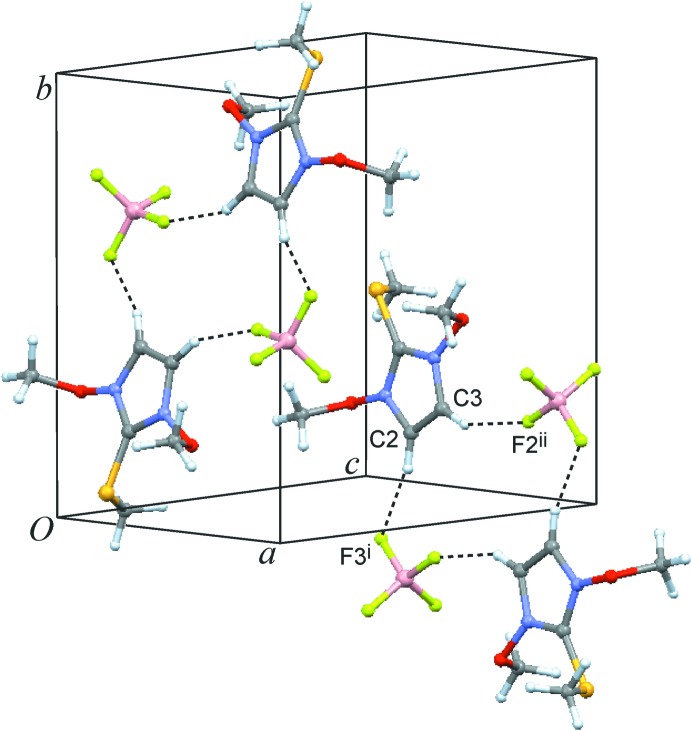
Crystal packing of compound **1**. Dashed lines represent the shortest inter­molecular C—H⋯F inter­actions. [Symmetry codes: (i) −*x* + 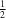, *y* − 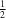, −*z* + 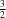; (ii) *x* + 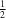, −*y* + 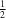, *z* + 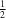.]

**Figure 5:**
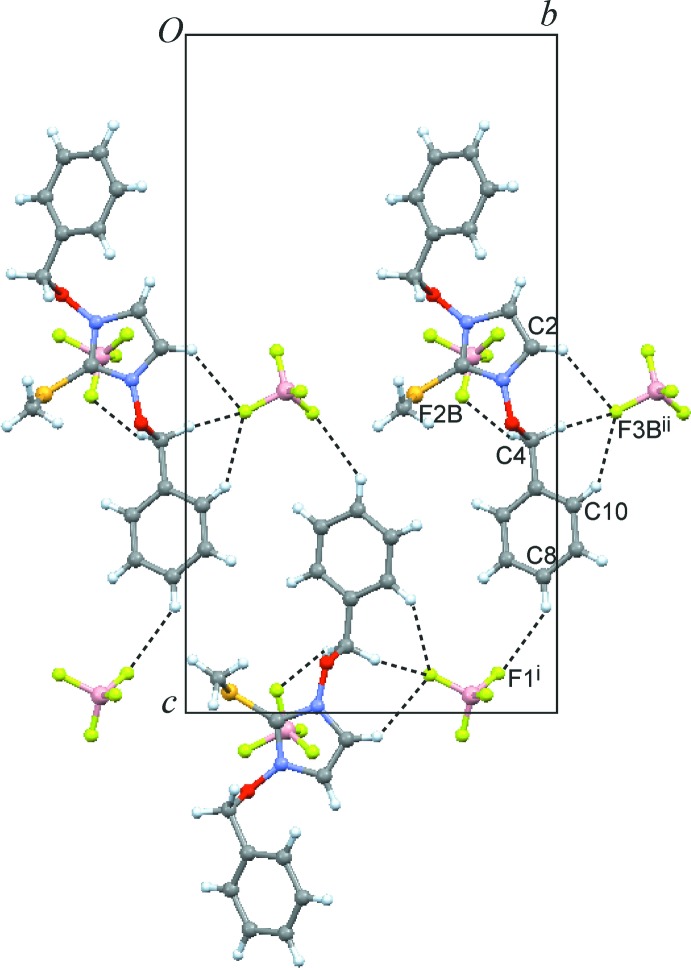
Crystal packing of compound **2**, viewed along the *a* axis. Dashed lines represent the shortest inter­molecular C—H⋯F inter­actions involving the disorder component *B* of the tetra­fluorido­borate anion. [Symmetry codes: (i) −*x* + 1, *y*, *z* + 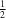; (ii) −*x* + 1, *y* + 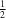, −*z* + 1.]

Both structures display multiple C—H⋯F—B contacts which cross-link the ion pairs and result in three-dimensional networks (Tables 1 ▸ and 2 ▸). In the crystal structure of **1**, the two most important of these inter­actions, C2—H2⋯F3(−*x* + 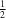, *y* − 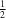, −*z* + 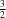) and C3—H3⋯F2(*x* + 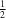, −*y* + 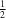, *z* + 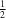), involve the two imidazole CH groups and yield a substructure with an 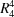
(14) motif (Etter *et al.*, 1990 ▸; Bernstein *et al.*, 1995 ▸) (Fig. 4 ▸). Moreover, the two meth­oxy groups are involved in this type of hydrogen bonding, albeit with weaker strength as can be seen in the longer H⋯F contacts (Table 1 ▸). In the di(benz­yloxy) salt **2**, each of the three disorder components of the anion gives rise to a specific set of C—H⋯F—B contacts. Fig. 5 ▸ shows the most significant inter­actions for one of the BF_4_^−^ components, which also involves one heterocyclic hydrogen (H2) as well as both methyl­ene (H4*A*, H4*B*; H11*A*) and two aromatic hydrogen atoms (H8, H10) of the cation. In contrast to **1**, the methyl­sulfanyl group (H18*C*) is also involved in hydrogen-bonding inter­actions.

## Database survey   

In addition to the classic 1,3-di­methyl­imidazolium-2-methyl­sulfanylimidazolium iodide (Williams *et al.*, 1994 ▸), the Cambridge Structural Database (Version 5.41 November 2019; Groom *et al.*, 2016 ▸) comprises a number of more unusual representatives such as very bulky 1,3-diaryl-2-phenyl­thio­imidazolium (Inés *et al.*, 2010 ▸) and 1,3-diaryl-2-methyl­sulfanylimidazolium salts (Liu *et al.*, 2017 ▸). These compounds are suitable precursors for the generation of exotic *N*-heterocyclic carbene–chalcogen cations.

Noteworthy is also the structure of a stabilized imidazoline-2-thione methyl­ide (Arduengo & Burgess, 1976 ▸). The attachment of a fluorine-containing group to a given mol­ecule may enhance certain properties and therefore widen the range of potential applications. For example, salts bearing S—CF_3_ groups (Mizuta *et al.*, 2016 ▸) have been found to be effective electrophilic phase-transfer catalysts. Additionally, the introduction of perfluoro­alkyl­thio groups (Hummel *et al.*, 2017 ▸) resulted in improved surfactant properties.

## Synthesis and crystallization   

**1,3-Dimeth­oxy-2-methyl­sulfanylimidazolium tetra­fluorido­borate (1):** Tri­methyl­oxonium tetra­fluorido­borate (0.51 g, 3.44 mmol) was added to a solution of 1,3-di­meth­oxy­imidazoline-2-thione (0.50 g, 3.12 mmol) in CH_2_Cl_2_ (20 ml). The mixture was stirred for 18 h at room temperature, then the solvent was evaporated. The residue was dissolved in EtOH (3 ml) and cooled at 277 K, forming colourless single crystals. The crystalline product was filtered, washed with Et2O (2 × 5 ml) and dried. Yield: 0.53 g, m.p. 363 K. ^1^H NMR (300 MHz, DMSO-*d*_6_): δ 2.72 (*s*, 3H), 4.25 (*s*, 6H), 8.43 (*s*, 2H) ppm ^13^C NMR (75 MHz, DMSO-*d*_6_): δ 16.3, 68.9 (2C), 118.0 (2C), 135.7 ppm IR (neat): ν 3153 (*m*), 3134 (*m*), 1552 (*m*), 1445 (*m*), 1287 (*w*), 1043 (*vs*), 1018 (*vs*), 937 (*s*), 754 (*s*), 734 (*s*), 689 (*m*), 672 (*m*), 619 (*m*), 520 (*s*) cm^−1^.

**1,3-Di(benz­yloxy)-2-methyl­sulfanylimidazolium tetra­fluor­ido­borate (2):** Tri­methyl­oxonium tetra­fluorido­borate (0.26 g, 1.7 mmol) was added to a solution of 1,3-di(benz­yloxy)imidazoline-2-thione (0.51 g, 1.6 mmol) in CH_2_Cl_2_ (8 ml) in a Teflon test tube under argon. The mixture was stirred for 3 d at room temperature, then the solvent was evaporated. The residue was dissolved in MeOH (15 ml), precipitated with Et_2_O (15 ml), filtered, washed with Et_2_O and dried to yield a colourless powder. Single crystals were obtained by slow evaporation from an MeOH solution: Yield 0.42 g (62%), m.p. 407 K. ^1^H NMR (300 MHz, DMSO-*d*_6_): δ 2.54 (*s*, 3H), 5.45 (*s*, 4H), 7.49 (*s*, 10H), 8.36 (*s*, 2H) ppm ^13^C NMR (75 MHz, DMSO-*d*_6_): δ 16.4, 83.1 (2C), 118.9 (2C), 128.9 (4C), 130.2 (2C), 130.4 (4C), 131.8 (2C), 136.8 ppm IR (neat): ν 3167 (*w*), 3137 (*w*), 1550 (*w*), 1492 (*w*), 1457 (*w*), 1384 (*w*), 1354 (*w*), 1214 (*w*), 1057 (*vs*), 1039 (*vs*), 907 (*m*), 873 (*m*), 844 (*m*), 773 (*s*), 739 (*s*), 699 (*s*), 671 (*s*), 575 (*m*), 499 (*m*) cm^−1^.

## Refinement   

Crystal data, data collection and structure refinement details are summarized in Table 3 ▸. All hydrogen atoms were identified in difference maps. Methyl H atoms were idealized and included as rigid groups allowed to rotate but not tip (C—H = 0.98 Å), and their *U*_iso_ parameters were set to 1.5 *U*_eq_(C) of the parent carbon atom. H atoms bonded to secondary carbon atoms (C—H = 0.99 Å), and H atoms bonded to C atoms in aromatic rings (C—H = 0.95 Å) were positioned geometrically and refined with *U*_iso_ set to 1.2 *U*_eq_(C) of the parent carbon atom.

The structure of **2** displays disorder of the tetra­fluorido­borate ion involving three distinct components. Therefore, distance restraints were applied for all chemically equivalent B—F and F⋯F distances and restraints on displacement parameters of the F atoms affected by disorder were applied.

## Supplementary Material

Crystal structure: contains datablock(s) 1, 2, global. DOI: 10.1107/S2056989020003643/wm5549sup1.cif

Structure factors: contains datablock(s) 1. DOI: 10.1107/S2056989020003643/wm55491sup2.hkl

Structure factors: contains datablock(s) 2. DOI: 10.1107/S2056989020003643/wm55492sup3.hkl

Supporting information file. DOI: 10.1107/S2056989020003643/wm55491sup4.cml

CCDC references: 1989707, 1989706

Additional supporting information:  crystallographic information; 3D view; checkCIF report

## Figures and Tables

**Table 1:** Hydrogen-bond geometry (Å, °) for **1** [Chem scheme1]

*D*—H⋯*A*	*D*—H	H⋯*A*	*D*⋯*A*	*D*—H⋯*A*
C2—H2⋯F3^i^	0.95	2.26	3.137 (3)	154
C2—H2⋯F2^i^	0.95	2.51	3.361 (3)	150
C3—H3⋯F2^ii^	0.95	2.33	3.207 (3)	153
C4—H4*B*⋯F3^iii^	0.98	2.61	3.408 (3)	139
C4—H4*B*⋯F4^iii^	0.98	2.52	3.487 (3)	168
C4—H4*C*⋯F2	0.98	2.46	3.418 (3)	167
C5—H5*A*⋯F3	0.98	2.54	3.519 (3)	177

Symmetry codes: (i) 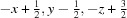; (ii) 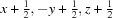; (iii) 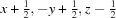.

**Table 2:** Hydrogen-bond geometry (Å, °) for **2** [Chem scheme1]

*D*—H⋯*A*	*D*—H	H⋯*A*	*D*⋯*A*	*D*—H⋯*A*
C2—H2⋯F2*A* ^i^	0.95	2.28	3.200 (13)	162
C2—H2⋯F3*B* ^i^	0.95	2.55	3.302 (14)	136
C2—H2⋯F2*C* ^i^	0.95	2.07	2.936 (10)	150
C4—H4*B*⋯F2*B*	0.99	2.52	3.472 (17)	162
C4—H4*A*⋯F3*B* ^i^	0.99	2.50	3.183 (13)	126
C4—H4*A*⋯F2*C* ^i^	0.99	2.56	3.39 (3)	141
C4—H4*B*⋯F4*C*	0.99	2.61	3.522 (19)	153
C8—H8⋯F1^ii^	0.95	2.54	3.317 (4)	139
C10—H10⋯F3*B* ^i^	0.95	2.46	3.282 (12)	145
C11—H11*A*⋯F3*A* ^iii^	0.99	2.27	3.247 (13)	167
C11—H11*A*⋯F3*C* ^iii^	0.99	2.40	3.279 (15)	148
C18—H18*C*⋯F3*A* ^iii^	0.98	2.62	3.409 (14)	138

Symmetry codes: (i) 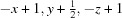; (ii) 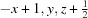; (iii) 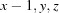.

**Table 3:** Experimental details

	**1**	**2**
Crystal data		
Chemical formula	C_6_H_11_N_2_O_2_S^+^·BF_4_^−^	C_18_H_19_N_2_O_2_S^+^·BF_4_^−^
*M*_r_	262.04	414.22
Crystal system, space group	Monoclinic, *P*2_1_/*n*	Orthorhombic, *P* *c*2_1_ *b*
Temperature (K)	173	173
*a*, *b*, *c* (Å)	8.1049 (7), 11.6979 (10), 12.0810 (12)	7.9117 (3), 11.4760 (4), 20.9659 (7)
α, β, γ (°)	90, 90.069 (9), 90	90, 90, 90
*V* (Å^3^)	1145.40 (18)	1903.59 (12)
*Z*	4	4
Radiation type	Mo *K*α	Mo *K*α
μ (mm^−1^)	0.32	0.22
Crystal size (mm)	0.20 × 0.14 × 0.12	0.44 × 0.36 × 0.12

Data collection		
Diffractometer	Rigaku Oxford Diffraction Xcalibur, Ruby, Gemini ultra	Rigaku Oxford Diffraction Xcalibur, Ruby, Gemini ultra
Absorption correction	Multi-scan (*CrysAlis PRO*; Rigaku OD, 2015 ▸)	Multi-scan (*CrysAlis PRO*; Rigaku OD, 2015 ▸)
*T*_min_, *T*_max_	0.973, 1	0.936, 1
No. of measured, independent and observed [*I* > 2σ(*I*)] reflections	8186, 2175, 1640	11654, 3555, 3293
*R*_int_	0.041	0.029
(sin θ/λ)_max_ (Å^−1^)	0.610	0.610

Refinement		
*R*[*F* ^2^ > 2σ(*F* ^2^)], *wR*(*F* ^2^), *S*	0.037, 0.088, 1.04	0.030, 0.067, 1.06
No. of reflections	2175	3555
No. of parameters	148	311
No. of restraints	0	368
H-atom treatment	H-atom parameters constrained	H-atom parameters constrained
Δρ_max_, Δρ_min_ (e Å^−3^)	0.25, −0.26	0.18, −0.23
Absolute structure	–	Flack *x* determined using 1454 quotients [(*I* ^+^)−(*I* ^−^)]/[(*I* ^+^)+(*I* ^−^)] (Parsons *et al.*, 2013 ▸).
Absolute structure parameter	–	0.02 (3)

Computer programs: *CrysAlis PRO* (Rigaku OD, 2015 ▸), *SIR2002* (Burla *et al.*, 2003 ▸), *SHELXL2014/6* (Sheldrick, 2015 ▸), *XP*/*SHELXTL* (Sheldrick, 2008 ▸), *Mercury* (Macrae *et al.*, 2020 ▸) and *publCIF* (Westrip, 2010 ▸).

## supplementary crystallographic information

### 1,3-Dimethoxy-2-(methylsulfanyl)imidazolium tetrafluoridoborate (1) Crystal data

**Table:**

C_6_H_11_N_2_O_2_S^+^·BF_4_^−^	*F*(000) = 536
*M**_r_* = 262.04	*D*_x_ = 1.520 Mg m^−^^3^
Monoclinic, *P*2_1_/*n*	Mo *K*α radiation, λ = 0.71073 Å
*a* = 8.1049 (7) Å	Cell parameters from 1882 reflections
*b* = 11.6979 (10) Å	θ = 4.5–23.4°
*c* = 12.0810 (12) Å	µ = 0.32 mm^−^^1^
β = 90.069 (9)°	*T* = 173 K
*V* = 1145.40 (18) Å^3^	Block, colourless
*Z* = 4	0.20 × 0.14 × 0.12 mm

### 1,3-Dimethoxy-2-(methylsulfanyl)imidazolium tetrafluoridoborate (1) Data collection

**Table:**

Rigaku Oxford Diffraction Xcalibur, Ruby, Gemini ultra diffractometer	2175 independent reflections
Radiation source: fine-focus sealed X-ray tube, Enhance (Mo) X-ray Source	1640 reflections with *I* > 2σ(*I*)
Graphite monochromator	*R*_int_ = 0.041
Detector resolution: 10.3575 pixels mm^-1^	θ_max_ = 25.7°, θ_min_ = 3.4°
ω scans	*h* = −9→9
Absorption correction: multi-scan (CrysAlisPro; Rigaku OD, 2015)	*k* = −14→14
*T*_min_ = 0.973, *T*_max_ = 1	*l* = −11→14
8186 measured reflections	

### 1,3-Dimethoxy-2-(methylsulfanyl)imidazolium tetrafluoridoborate (1) Refinement

**Table:**

Refinement on *F*^2^	Primary atom site location: structure-invariant direct methods
Least-squares matrix: full	Secondary atom site location: difference Fourier map
*R*[*F*^2^ > 2σ(*F*^2^)] = 0.037	Hydrogen site location: inferred from neighbouring sites
*wR*(*F*^2^) = 0.088	H-atom parameters constrained
*S* = 1.04	*w* = 1/[σ^2^(*F*_o_^2^) + (0.0305*P*)^2^ + 0.4296*P*] where *P* = (*F*_o_^2^ + 2*F*_c_^2^)/3
2175 reflections	(Δ/σ)_max_ < 0.001
148 parameters	Δρ_max_ = 0.25 e Å^−^^3^
0 restraints	Δρ_min_ = −0.26 e Å^−^^3^

### 1,3-Dimethoxy-2-(methylsulfanyl)imidazolium tetrafluoridoborate (1) Special details

**Table:**

Geometry. All esds (except the esd in the dihedral angle between two l.s. planes) are estimated using the full covariance matrix. The cell esds are taken into account individually in the estimation of esds in distances, angles and torsion angles; correlations between esds in cell parameters are only used when they are defined by crystal symmetry. An approximate (isotropic) treatment of cell esds is used for estimating esds involving l.s. planes.

### 1,3-Dimethoxy-2-(methylsulfanyl)imidazolium tetrafluoridoborate (1) Fractional atomic coordinates and isotropic or equivalent isotropic displacement parameters (Å^2^)

**Table:**

	*x*	*y*	*z*	*U*_iso_*/*U*_eq_	
S1	0.41330 (7)	0.47008 (5)	0.72979 (5)	0.03744 (19)	
F1	0.08382 (16)	0.25059 (12)	0.80034 (13)	0.0477 (4)	
F2	−0.01740 (16)	0.36253 (12)	0.66193 (10)	0.0439 (4)	
F3	−0.0285 (2)	0.42160 (11)	0.83926 (11)	0.0516 (4)	
F4	−0.18980 (16)	0.27590 (12)	0.78296 (13)	0.0514 (4)	
O1	0.46146 (18)	0.22793 (13)	0.61505 (12)	0.0344 (4)	
O2	0.45275 (18)	0.38275 (13)	0.97279 (12)	0.0340 (4)	
N2	0.4484 (2)	0.30437 (14)	0.88842 (14)	0.0255 (4)	
N1	0.4543 (2)	0.23500 (14)	0.72783 (14)	0.0251 (4)	
C1	0.4420 (2)	0.33444 (17)	0.78163 (17)	0.0246 (5)	
C2	0.4692 (2)	0.14498 (18)	0.79882 (19)	0.0304 (5)	
H2	0.4810	0.0667	0.7796	0.036*	
C3	0.4638 (2)	0.18919 (18)	0.90157 (19)	0.0301 (5)	
H3	0.4696	0.1484	0.9695	0.036*	
C5	0.2956 (3)	0.3860 (2)	1.0302 (2)	0.0389 (6)	
H5A	0.2067	0.3988	0.9766	0.058*	
H5B	0.2965	0.4482	1.0845	0.058*	
H5C	0.2775	0.3131	1.0683	0.058*	
C6	0.6002 (3)	0.4910 (2)	0.6533 (2)	0.0477 (7)	
H6A	0.6951	0.4823	0.7029	0.072*	
H6B	0.6007	0.5680	0.6213	0.072*	
H6C	0.6069	0.4343	0.5938	0.072*	
B1	−0.0377 (3)	0.3266 (2)	0.7711 (2)	0.0276 (6)	
C4	0.3017 (3)	0.1968 (2)	0.5703 (2)	0.0471 (7)	
H4A	0.2681	0.1226	0.6004	0.071*	
H4B	0.3088	0.1917	0.4895	0.071*	
H4C	0.2203	0.2549	0.5908	0.071*	

### 1,3-Dimethoxy-2-(methylsulfanyl)imidazolium tetrafluoridoborate (1) Atomic displacement parameters (Å^2^)

**Table:**

	*U*^11^	*U*^22^	*U*^33^	*U*^12^	*U*^13^	*U*^23^
S1	0.0346 (3)	0.0294 (3)	0.0484 (4)	0.0073 (2)	0.0086 (3)	0.0077 (3)
F1	0.0337 (8)	0.0490 (9)	0.0605 (10)	0.0094 (6)	−0.0015 (7)	0.0051 (7)
F2	0.0500 (8)	0.0562 (9)	0.0254 (7)	0.0007 (7)	0.0062 (6)	0.0000 (6)
F3	0.0889 (12)	0.0336 (8)	0.0323 (8)	−0.0033 (7)	0.0035 (7)	−0.0043 (6)
F4	0.0278 (7)	0.0643 (10)	0.0621 (10)	−0.0099 (7)	−0.0001 (7)	0.0120 (8)
O1	0.0272 (8)	0.0512 (10)	0.0247 (9)	−0.0011 (7)	0.0056 (6)	−0.0087 (7)
O2	0.0307 (8)	0.0409 (9)	0.0305 (9)	−0.0101 (7)	0.0022 (7)	−0.0140 (7)
N2	0.0237 (9)	0.0262 (9)	0.0266 (10)	−0.0035 (7)	−0.0003 (7)	−0.0059 (8)
N1	0.0219 (9)	0.0292 (10)	0.0242 (10)	−0.0009 (7)	0.0008 (7)	−0.0048 (8)
C1	0.0183 (10)	0.0277 (11)	0.0276 (12)	0.0007 (9)	0.0019 (9)	−0.0019 (10)
C2	0.0243 (11)	0.0254 (11)	0.0415 (14)	−0.0001 (9)	−0.0022 (10)	0.0010 (10)
C3	0.0277 (11)	0.0289 (12)	0.0337 (14)	−0.0020 (9)	−0.0052 (10)	0.0052 (10)
C5	0.0343 (13)	0.0493 (15)	0.0331 (13)	0.0004 (11)	0.0069 (10)	−0.0069 (11)
C6	0.0413 (15)	0.0406 (14)	0.0613 (18)	0.0012 (12)	0.0150 (13)	0.0150 (13)
B1	0.0253 (13)	0.0294 (13)	0.0282 (14)	−0.0015 (11)	0.0014 (10)	−0.0017 (11)
C4	0.0339 (13)	0.0779 (19)	0.0294 (14)	−0.0056 (13)	−0.0041 (11)	−0.0124 (13)

### 1,3-Dimethoxy-2-(methylsulfanyl)imidazolium tetrafluoridoborate (1) Geometric parameters (Å, º)

**Table:**

S1—C1	1.722 (2)	N1—C2	1.363 (3)
S1—C6	1.792 (2)	C2—C3	1.346 (3)
F1—B1	1.373 (3)	C2—H2	0.9500
F2—B1	1.394 (3)	C3—H3	0.9500
F3—B1	1.385 (3)	C5—H5A	0.9800
F4—B1	1.376 (3)	C5—H5B	0.9800
O1—N1	1.366 (2)	C5—H5C	0.9800
O1—C4	1.449 (3)	C6—H6A	0.9800
O2—N2	1.372 (2)	C6—H6B	0.9800
O2—C5	1.451 (3)	C6—H6C	0.9800
N2—C1	1.338 (3)	C4—H4A	0.9800
N2—C3	1.362 (3)	C4—H4B	0.9800
N1—C1	1.336 (3)	C4—H4C	0.9800
			
C1—S1—C6	101.52 (10)	O2—C5—H5C	109.5
N1—O1—C4	110.37 (15)	H5A—C5—H5C	109.5
N2—O2—C5	110.52 (15)	H5B—C5—H5C	109.5
C1—N2—C3	112.08 (17)	S1—C6—H6A	109.5
C1—N2—O2	122.80 (17)	S1—C6—H6B	109.5
C3—N2—O2	124.90 (17)	H6A—C6—H6B	109.5
C1—N1—C2	111.90 (18)	S1—C6—H6C	109.5
C1—N1—O1	122.75 (17)	H6A—C6—H6C	109.5
C2—N1—O1	125.22 (17)	H6B—C6—H6C	109.5
N1—C1—N2	103.73 (17)	F1—B1—F4	109.66 (19)
N1—C1—S1	129.46 (17)	F1—B1—F3	109.20 (19)
N2—C1—S1	126.73 (16)	F4—B1—F3	109.36 (18)
C3—C2—N1	106.29 (19)	F1—B1—F2	110.68 (18)
C3—C2—H2	126.9	F4—B1—F2	109.58 (19)
N1—C2—H2	126.9	F3—B1—F2	108.32 (18)
C2—C3—N2	106.00 (19)	O1—C4—H4A	109.5
C2—C3—H3	127.0	O1—C4—H4B	109.5
N2—C3—H3	127.0	H4A—C4—H4B	109.5
O2—C5—H5A	109.5	O1—C4—H4C	109.5
O2—C5—H5B	109.5	H4A—C4—H4C	109.5
H5A—C5—H5B	109.5	H4B—C4—H4C	109.5

### 1,3-Dimethoxy-2-(methylsulfanyl)imidazolium tetrafluoridoborate (1) Hydrogen-bond geometry (Å, º)

**Table:**

*D*—H···*A*	*D*—H	H···*A*	*D*···*A*	*D*—H···*A*
C2—H2···F3^i^	0.95	2.26	3.137 (3)	154
C2—H2···F2^i^	0.95	2.51	3.361 (3)	150
C3—H3···F2^ii^	0.95	2.33	3.207 (3)	153
C4—H4*B*···F3^iii^	0.98	2.61	3.408 (3)	139
C4—H4*B*···F4^iii^	0.98	2.52	3.487 (3)	168
C4—H4*C*···F2	0.98	2.46	3.418 (3)	167
C5—H5*A*···F3	0.98	2.54	3.519 (3)	177

Symmetry codes: (i) −*x*+1/2, *y*−1/2, −*z*+3/2; (ii) *x*+1/2, −*y*+1/2, *z*+1/2; (iii) *x*+1/2, −*y*+1/2, *z*−1/2.

### 1,3-Dibenzyloxy-2-(methylsulfanyl)imidazolium tetrafluoridoborate (2) Crystal data

**Table:**

C_18_H_19_N_2_O_2_S^+^·BF_4_^−^	*F*(000) = 856
*M**_r_* = 414.22	*D*_x_ = 1.445 Mg m^−^^3^
Orthorhombic, *P**c*2_1_*b*	Mo *K*α radiation, λ = 0.71073 Å
Hall symbol: P -2bc -2c	Cell parameters from 6176 reflections
*a* = 7.9117 (3) Å	θ = 3.1–28.6°
*b* = 11.4760 (4) Å	µ = 0.22 mm^−^^1^
*c* = 20.9659 (7) Å	*T* = 173 K
*V* = 1903.59 (12) Å^3^	Plate, colourless
*Z* = 4	0.44 × 0.36 × 0.12 mm

### 1,3-Dibenzyloxy-2-(methylsulfanyl)imidazolium tetrafluoridoborate (2) Data collection

**Table:**

Rigaku Oxford Diffraction Xcalibur, Ruby, Gemini ultra diffractometer	3555 independent reflections
Graphite monochromator	3293 reflections with *I* > 2σ(*I*)
Detector resolution: 10.3575 pixels mm^-1^	*R*_int_ = 0.029
ω scans	θ_max_ = 25.7°, θ_min_ = 3.2°
Absorption correction: multi-scan (CrysAlisPro; Rigaku OD, 2015)	*h* = −9→8
*T*_min_ = 0.936, *T*_max_ = 1	*k* = −13→13
11654 measured reflections	*l* = −19→25

### 1,3-Dibenzyloxy-2-(methylsulfanyl)imidazolium tetrafluoridoborate (2) Refinement

**Table:**

Refinement on *F*^2^	Secondary atom site location: difference Fourier map
Least-squares matrix: full	Hydrogen site location: inferred from neighbouring sites
*R*[*F*^2^ > 2σ(*F*^2^)] = 0.030	H-atom parameters constrained
*wR*(*F*^2^) = 0.067	*w* = 1/[σ^2^(*F*_o_^2^) + (0.031*P*)^2^ + 0.3041*P*] where *P* = (*F*_o_^2^ + 2*F*_c_^2^)/3
*S* = 1.06	(Δ/σ)_max_ = 0.007
3555 reflections	Δρ_max_ = 0.18 e Å^−^^3^
311 parameters	Δρ_min_ = −0.22 e Å^−^^3^
368 restraints	Absolute structure: Flack *x* determined using 1454 quotients [(*I*^+^)-(*I*^-^)]/[(*I*^+^)+(*I*^-^)] (Parsons *et al.*, 2013).
Primary atom site location: structure-invariant direct methods	Absolute structure parameter: 0.02 (3)

### 1,3-Dibenzyloxy-2-(methylsulfanyl)imidazolium tetrafluoridoborate (2) Special details

**Table:**

Geometry. All esds (except the esd in the dihedral angle between two l.s. planes) are estimated using the full covariance matrix. The cell esds are taken into account individually in the estimation of esds in distances, angles and torsion angles; correlations between esds in cell parameters are only used when they are defined by crystal symmetry. An approximate (isotropic) treatment of cell esds is used for estimating esds involving l.s. planes.

### 1,3-Dibenzyloxy-2-(methylsulfanyl)imidazolium tetrafluoridoborate (2) Fractional atomic coordinates and isotropic or equivalent isotropic displacement parameters (Å^2^)

**Table:**

	*x*	*y*	*z*	*U*_iso_*/*U*_eq_	Occ. (<1)
B1	0.7773 (4)	0.7700 (3)	0.47458 (15)	0.0309 (7)	
F1	0.6678 (2)	0.84120 (18)	0.44219 (10)	0.0588 (6)	
F2B	0.719 (2)	0.7454 (15)	0.5332 (6)	0.071 (6)	0.341 (5)
F3B	0.781 (3)	0.6596 (8)	0.4439 (7)	0.072 (5)	0.341 (5)
F4B	0.9356 (13)	0.8091 (14)	0.4735 (12)	0.083 (9)	0.341 (5)
F2C	0.7136 (18)	0.6688 (14)	0.4845 (18)	0.093 (7)	0.238 (4)
F3C	0.9288 (16)	0.7723 (19)	0.4449 (8)	0.060 (6)	0.238 (4)
F4C	0.803 (3)	0.834 (2)	0.5316 (6)	0.089 (6)	0.238 (4)
F2A	0.6947 (13)	0.7094 (11)	0.5212 (5)	0.044 (3)	0.421 (4)
F3A	0.849 (2)	0.6901 (17)	0.4354 (6)	0.126 (8)	0.421 (4)
F4A	0.9031 (14)	0.8315 (12)	0.5042 (8)	0.076 (5)	0.421 (4)
S1	0.33131 (8)	0.61824 (6)	0.53046 (3)	0.02961 (17)	
O2	0.3430 (2)	0.66702 (16)	0.38403 (8)	0.0283 (4)	
O1	0.2637 (2)	0.87919 (17)	0.57345 (8)	0.0273 (4)	
N1	0.2835 (2)	0.85434 (19)	0.50947 (9)	0.0223 (5)	
N2	0.3093 (2)	0.75797 (18)	0.42440 (10)	0.0214 (5)	
C1	0.3058 (3)	0.7453 (2)	0.48800 (12)	0.0208 (5)	
C3	0.2924 (3)	0.8725 (2)	0.40683 (12)	0.0256 (6)	
H3	0.2937	0.9028	0.3647	0.031*	
C12	0.1490 (3)	0.6677 (2)	0.29313 (12)	0.0261 (6)	
C9	0.2992 (3)	1.0534 (3)	0.75895 (14)	0.0350 (7)	
H9	0.2491	1.1218	0.7762	0.042*	
C10	0.3228 (3)	1.0439 (3)	0.69382 (13)	0.0304 (6)	
H10	0.2893	1.1057	0.6665	0.036*	
C5	0.3952 (3)	0.9444 (2)	0.66841 (12)	0.0280 (6)	
C6	0.4421 (4)	0.8550 (3)	0.70894 (13)	0.0342 (7)	
H6	0.4907	0.7860	0.6919	0.041*	
C11	0.1862 (3)	0.6160 (3)	0.35716 (12)	0.0314 (6)	
H11A	0.0904	0.6312	0.3864	0.038*	
H11B	0.1995	0.5305	0.3530	0.038*	
C15	0.0978 (4)	0.7695 (3)	0.17451 (14)	0.0389 (7)	
H15	0.0811	0.8047	0.1340	0.047*	
C17	0.0445 (3)	0.7640 (3)	0.28694 (13)	0.0331 (6)	
H17	−0.0103	0.7953	0.3234	0.040*	
C2	0.2736 (3)	0.9333 (2)	0.46104 (12)	0.0252 (6)	
H2	0.2567	1.0150	0.4649	0.030*	
C7	0.4189 (4)	0.8652 (3)	0.77389 (14)	0.0392 (7)	
H7	0.4521	0.8034	0.8013	0.047*	
C8	0.3482 (4)	0.9641 (3)	0.79913 (14)	0.0369 (7)	
H8	0.3329	0.9712	0.8439	0.044*	
C4	0.4199 (4)	0.9319 (3)	0.59824 (13)	0.0405 (8)	
H4A	0.4396	1.0090	0.5783	0.049*	
H4B	0.5183	0.8812	0.5891	0.049*	
C13	0.2265 (3)	0.6217 (3)	0.23927 (12)	0.0318 (6)	
H13	0.2976	0.5554	0.2429	0.038*	
C18	0.1150 (4)	0.5878 (3)	0.55032 (17)	0.0498 (9)	
H18A	0.0666	0.6545	0.5731	0.075*	
H18B	0.1098	0.5185	0.5775	0.075*	
H18C	0.0506	0.5738	0.5111	0.075*	
C14	0.2001 (4)	0.6726 (3)	0.18030 (14)	0.0396 (7)	
H14	0.2527	0.6406	0.1435	0.047*	
C16	0.0199 (4)	0.8148 (3)	0.22780 (14)	0.0389 (7)	
H16	−0.0511	0.8812	0.2239	0.047*	

### 1,3-Dibenzyloxy-2-(methylsulfanyl)imidazolium tetrafluoridoborate (2) Atomic displacement parameters (Å^2^)

**Table:**

	*U*^11^	*U*^22^	*U*^33^	*U*^12^	*U*^13^	*U*^23^
B1	0.0229 (16)	0.040 (2)	0.0298 (17)	0.0004 (14)	−0.0001 (12)	0.0065 (16)
F1	0.0409 (11)	0.0542 (13)	0.0813 (14)	−0.0050 (9)	−0.0231 (9)	0.0296 (11)
F2B	0.102 (13)	0.081 (13)	0.029 (4)	0.002 (9)	0.017 (5)	−0.004 (6)
F3B	0.136 (16)	0.027 (4)	0.052 (7)	−0.007 (7)	−0.004 (8)	−0.014 (4)
F4B	0.025 (4)	0.051 (9)	0.17 (3)	0.002 (4)	0.011 (10)	0.043 (14)
F2C	0.066 (9)	0.037 (10)	0.17 (2)	−0.010 (7)	−0.002 (14)	0.053 (14)
F3C	0.031 (7)	0.087 (16)	0.061 (8)	0.015 (7)	0.020 (5)	−0.001 (8)
F4C	0.092 (12)	0.138 (17)	0.037 (6)	0.040 (11)	−0.013 (7)	−0.024 (8)
F2A	0.039 (3)	0.038 (6)	0.055 (7)	−0.012 (4)	0.007 (4)	0.014 (5)
F3A	0.101 (10)	0.21 (2)	0.071 (6)	0.089 (12)	0.018 (7)	−0.037 (11)
F4A	0.044 (7)	0.073 (8)	0.110 (11)	−0.038 (7)	−0.047 (7)	0.047 (7)
S1	0.0245 (3)	0.0282 (3)	0.0362 (4)	0.0029 (3)	0.0005 (2)	0.0108 (3)
O2	0.0270 (9)	0.0310 (10)	0.0269 (9)	0.0062 (8)	−0.0021 (7)	−0.0098 (8)
O1	0.0244 (9)	0.0357 (11)	0.0217 (9)	−0.0087 (8)	0.0059 (6)	−0.0073 (8)
N1	0.0218 (10)	0.0238 (12)	0.0211 (11)	−0.0014 (9)	0.0033 (8)	−0.0032 (9)
N2	0.0207 (10)	0.0211 (12)	0.0225 (11)	0.0024 (9)	0.0010 (8)	−0.0032 (9)
C1	0.0180 (11)	0.0229 (14)	0.0214 (13)	−0.0012 (10)	0.0001 (9)	0.0016 (11)
C3	0.0226 (12)	0.0271 (14)	0.0271 (13)	−0.0004 (11)	0.0009 (9)	0.0071 (13)
C12	0.0228 (12)	0.0268 (14)	0.0285 (13)	−0.0044 (11)	−0.0056 (10)	−0.0050 (12)
C9	0.0378 (15)	0.0281 (16)	0.0390 (16)	0.0001 (12)	0.0049 (12)	−0.0099 (14)
C10	0.0317 (14)	0.0248 (15)	0.0345 (15)	−0.0033 (12)	−0.0019 (11)	0.0027 (13)
C5	0.0228 (13)	0.0330 (15)	0.0281 (14)	−0.0099 (11)	0.0013 (10)	−0.0051 (12)
C6	0.0286 (14)	0.0287 (16)	0.0454 (17)	0.0007 (12)	−0.0035 (11)	−0.0099 (14)
C11	0.0370 (14)	0.0239 (13)	0.0332 (14)	−0.0047 (13)	−0.0049 (10)	−0.0033 (15)
C15	0.0431 (17)	0.0391 (18)	0.0345 (16)	−0.0097 (14)	−0.0149 (13)	0.0054 (14)
C17	0.0259 (13)	0.0358 (17)	0.0376 (16)	0.0009 (12)	−0.0036 (11)	−0.0079 (14)
C2	0.0213 (13)	0.0197 (13)	0.0346 (15)	−0.0018 (10)	0.0032 (10)	0.0007 (12)
C7	0.0406 (17)	0.0327 (17)	0.0443 (17)	−0.0056 (13)	−0.0148 (12)	0.0075 (16)
C8	0.0434 (18)	0.0428 (18)	0.0244 (15)	−0.0114 (14)	−0.0016 (12)	−0.0032 (13)
C4	0.0281 (14)	0.061 (2)	0.0327 (15)	−0.0200 (14)	0.0055 (12)	−0.0142 (15)
C13	0.0338 (14)	0.0274 (14)	0.0341 (15)	0.0017 (13)	−0.0056 (10)	−0.0108 (15)
C18	0.0327 (16)	0.042 (2)	0.074 (2)	0.0042 (14)	0.0209 (15)	0.0267 (17)
C14	0.0437 (17)	0.0448 (18)	0.0302 (15)	−0.0041 (15)	−0.0035 (13)	−0.0108 (14)
C16	0.0351 (16)	0.0323 (17)	0.049 (2)	0.0020 (13)	−0.0142 (13)	0.0023 (14)

### 1,3-Dibenzyloxy-2-(methylsulfanyl)imidazolium tetrafluoridoborate (2) Geometric parameters (Å, º)

**Table:**

B1—F2C	1.283 (11)	C9—H9	0.9500
B1—F4B	1.330 (10)	C10—C5	1.384 (4)
B1—F2B	1.342 (11)	C10—H10	0.9500
B1—F3C	1.350 (11)	C5—C6	1.383 (4)
B1—F3A	1.355 (10)	C5—C4	1.491 (4)
B1—F2A	1.367 (8)	C6—C7	1.379 (4)
B1—F4A	1.369 (9)	C6—H6	0.9500
B1—F1	1.371 (4)	C11—H11A	0.9900
B1—F4C	1.414 (12)	C11—H11B	0.9900
B1—F3B	1.422 (9)	C15—C16	1.378 (4)
S1—C1	1.721 (3)	C15—C14	1.381 (5)
S1—C18	1.795 (3)	C15—H15	0.9500
O2—N2	1.370 (3)	C17—C16	1.384 (4)
O2—C11	1.483 (3)	C17—H17	0.9500
O1—N1	1.380 (3)	C2—H2	0.9500
O1—C4	1.471 (3)	C7—C8	1.372 (5)
N1—C1	1.341 (3)	C7—H7	0.9500
N1—C2	1.363 (3)	C8—H8	0.9500
N2—C1	1.342 (3)	C4—H4A	0.9900
N2—C3	1.372 (3)	C4—H4B	0.9900
C3—C2	1.342 (4)	C13—C14	1.383 (4)
C3—H3	0.9500	C13—H13	0.9500
C12—C17	1.386 (4)	C18—H18A	0.9800
C12—C13	1.389 (4)	C18—H18B	0.9800
C12—C11	1.497 (4)	C18—H18C	0.9800
C9—C8	1.382 (4)	C14—H14	0.9500
C9—C10	1.383 (4)	C16—H16	0.9500
			
F4B—B1—F2B	114.1 (10)	C7—C6—C5	120.6 (3)
F2C—B1—F3C	116.1 (11)	C7—C6—H6	119.7
F3A—B1—F2A	106.7 (8)	C5—C6—H6	119.7
F3A—B1—F4A	108.8 (8)	O2—C11—C12	110.4 (2)
F2A—B1—F4A	106.6 (7)	O2—C11—H11A	109.6
F2C—B1—F1	111.8 (7)	C12—C11—H11A	109.6
F4B—B1—F1	112.7 (7)	O2—C11—H11B	109.6
F2B—B1—F1	111.3 (8)	C12—C11—H11B	109.6
F3C—B1—F1	108.8 (8)	H11A—C11—H11B	108.1
F3A—B1—F1	111.5 (6)	C16—C15—C14	119.7 (3)
F2A—B1—F1	110.8 (5)	C16—C15—H15	120.2
F4A—B1—F1	112.2 (6)	C14—C15—H15	120.2
F2C—B1—F4C	112.7 (11)	C16—C17—C12	120.3 (3)
F3C—B1—F4C	104.6 (10)	C16—C17—H17	119.9
F1—B1—F4C	101.7 (6)	C12—C17—H17	119.9
F4B—B1—F3B	105.9 (8)	C3—C2—N1	106.2 (2)
F2B—B1—F3B	103.5 (8)	C3—C2—H2	126.9
F1—B1—F3B	108.6 (7)	N1—C2—H2	126.9
C1—S1—C18	99.97 (13)	C8—C7—C6	120.3 (3)
N2—O2—C11	111.87 (18)	C8—C7—H7	119.8
N1—O1—C4	109.43 (17)	C6—C7—H7	119.8
C1—N1—C2	112.2 (2)	C7—C8—C9	119.5 (3)
C1—N1—O1	122.3 (2)	C7—C8—H8	120.2
C2—N1—O1	125.5 (2)	C9—C8—H8	120.2
C1—N2—O2	122.4 (2)	O1—C4—C5	106.1 (2)
C1—N2—C3	111.6 (2)	O1—C4—H4A	110.5
O2—N2—C3	125.7 (2)	C5—C4—H4A	110.5
N1—C1—N2	103.6 (2)	O1—C4—H4B	110.5
N1—C1—S1	129.23 (19)	C5—C4—H4B	110.5
N2—C1—S1	127.1 (2)	H4A—C4—H4B	108.7
C2—C3—N2	106.4 (2)	C14—C13—C12	120.0 (3)
C2—C3—H3	126.8	C14—C13—H13	120.0
N2—C3—H3	126.8	C12—C13—H13	120.0
C17—C12—C13	119.3 (3)	S1—C18—H18A	109.5
C17—C12—C11	121.2 (2)	S1—C18—H18B	109.5
C13—C12—C11	119.4 (2)	H18A—C18—H18B	109.5
C8—C9—C10	120.4 (3)	S1—C18—H18C	109.5
C8—C9—H9	119.8	H18A—C18—H18C	109.5
C10—C9—H9	119.8	H18B—C18—H18C	109.5
C9—C10—C5	120.1 (3)	C15—C14—C13	120.5 (3)
C9—C10—H10	120.0	C15—C14—H14	119.8
C5—C10—H10	120.0	C13—C14—H14	119.8
C6—C5—C10	119.1 (2)	C15—C16—C17	120.3 (3)
C6—C5—C4	120.0 (3)	C15—C16—H16	119.9
C10—C5—C4	120.9 (3)	C17—C16—H16	119.9
			
N1—O1—C4—C5	−174.2 (2)	N2—O2—C11—C12	95.5 (2)

### 1,3-Dibenzyloxy-2-(methylsulfanyl)imidazolium tetrafluoridoborate (2) Hydrogen-bond geometry (Å, º)

**Table:**

*D*—H···*A*	*D*—H	H···*A*	*D*···*A*	*D*—H···*A*
C2—H2···F2*A*^i^	0.95	2.28	3.200 (13)	162
C2—H2···F3*B*^i^	0.95	2.55	3.302 (14)	136
C2—H2···F2*C*^i^	0.95	2.07	2.936 (10)	150
C4—H4*B*···F2*B*	0.99	2.52	3.472 (17)	162
C4—H4*A*···F3*B*^i^	0.99	2.50	3.183 (13)	126
C4—H4*A*···F2*C*^i^	0.99	2.56	3.39 (3)	141
C4—H4*B*···F4*C*	0.99	2.61	3.522 (19)	153
C8—H8···F1^ii^	0.95	2.54	3.317 (4)	139
C10—H10···F3*B*^i^	0.95	2.46	3.282 (12)	145
C11—H11*A*···F3*A*^iii^	0.99	2.27	3.247 (13)	167
C11—H11*A*···F3*C*^iii^	0.99	2.40	3.279 (15)	148
C18—H18*C*···F3*A*^iii^	0.98	2.62	3.409 (14)	138

Symmetry codes: (i) −*x*+1, *y*+1/2, −*z*+1; (ii) −*x*+1, *y*, *z*+1/2; (iii) *x*−1, *y*, *z*.

## References

[bb1] Arduengo, A. J. & Burgess, E. M. (1976). *J. Am. Chem. Soc.* **98**, 5021–5023.

[bb2] Bernstein, J., Davis, R. E., Shimoni, L. & Chang, N.-L. (1995). *Angew. Chem. Int. Ed. Engl.* **34**, 1555–1573.

[bb3] Burla, M. C., Camalli, M., Carrozzini, B., Cascarano, G. L., Giacovazzo, C., Polidori, G. & Spagna, R. (2003). *J. Appl. Cryst.* **36**, 1103. 10.1107/s0108767399007205 10927316

[bb4] Etter, M. C., MacDonald, J. C. & Bernstein, J. (1990). *Acta Cryst.* B**46**, 256–262. 10.1107/s0108768189012929 2344397

[bb5] Fürstner, A., Alcarazo, M., Goddard, R. & Lehmann, C. W. (2008). *Angew. Chem. Int. Ed.* **47**, 3210–3214. 10.1002/anie.200705798 18348113

[bb6] Groom, C. R., Bruno, I. J., Lightfoot, M. P. & Ward, S. C. (2016). *Acta Cryst.* B**72**, 171–179. 10.1107/S2052520616003954 PMC4822653 27048719

[bb7] Guterman, R., Miao, H. & Antonietti, M. (2018). *J. Org. Chem.* **83**, 684–689. 10.1021/acs.joc.7b02631 29257693

[bb8] Hummel, M., Markiewicz, M., Stolte, S., Noisternig, M. E., Braun, D., Gelbrich, T., Griesser, U. J., Partl, G., Naier, B., Wurst, K., Krüger, B., Kopacka, H., Laus, G., Huppertz, H. & Schottenberger, H. (2017). *Green Chem.* **19**, 3225–3237.

[bb9] Inés, B., Holle, S., Goddard, R. & Alcarazo, M. (2010). *Angew. Chem. Int. Ed.* **49**, 8389–8391. 10.1002/anie.201004149 20886490

[bb10] Kuhn, N., Bohnen, H., Kratz, T. & Henkel, G. (1993). *Liebigs Ann. Chem.* pp. 1149–1151.

[bb11] Laus, G., Kahlenberg, V., Wurst, K., Müller, T., Kopacka, H. & Schottenberger, H. (2013). *Z. Naturforsch. Teil B*, **68**, 1239–1252.

[bb12] Laus, G., Kostner, M. E., Kahlenberg, V. & Schottenberger, H. (2016). *Z. Naturforsch. Teil B*, **71**, 997–1003.

[bb13] Link, H., Klötzer, W., Karpitschka, E. M., Montavon, M., Müssner, R. & Singewald, N. (1990). *Angew. Chem.* **102**, 559–560.

[bb14] Liu, L. L., Zhu, D., Cao, L. L. & Stephan, D. W. (2017). *Dalton Trans.* **46**, 3095–3099. 10.1039/c7dt00186j 28145552

[bb15] Macrae, C. F., Sovago, I., Cottrell, S. J., Galek, P. T. A., McCabe, P., Pidcock, E., Platings, M., Shields, G. P., Stevens, J. S., Towler, M. & Wood, P. A. (2020). *J. Appl. Cryst.* **53**, 226–235. 10.1107/S1600576719014092 PMC6998782 32047413

[bb16] Mizuta, S., Kitamura, K., Nishi, K., Hashimoto, R., Usui, T. & Chiba, K. (2016). *RSC Adv.* **6**, 43159–43162.

[bb17] Parsons, S., Flack, H. D. & Wagner, T. (2013). *Acta Cryst.* B**69**, 249–259. 10.1107/S2052519213010014 PMC3661305 23719469

[bb18] Patel, N., Arfeen, M., Sood, R., Khullar, S., Chakraborti, A. K., Mandal, S. K. & Bharatam, P. V. (2018). *Chem. Eur. J.* **24**, 6418–6425. 10.1002/chem.201705999 29504658

[bb19] Rigaku OD (2015). *CrysAlis PRO.* Rigaku Oxford Diffraction, Yarnton, England.

[bb20] Sheldrick, G. M. (2008). *Acta Cryst.* A**64**, 112–122. 10.1107/S0108767307043930 18156677

[bb21] Sheldrick, G. M. (2015). *Acta Cryst.* C**71**, 3–8.

[bb22] Westrip, S. P. (2010). *J. Appl. Cryst.* **43**, 920–925.

[bb23] Williams, D. J., Ly, T. A., Mudge, J. W., VanDerveer, D. & Jones, R. L. (1994). *Inorg. Chim. Acta*, **218**, 133–138.

